# Incorporation of *Citrus* Peel-Derived Bioactive Compounds into a Fish-Based Food Product: Effects on Quality, Antioxidant Potential, Microbial Safety and Sensory Attributes

**DOI:** 10.3390/foods15101741

**Published:** 2026-05-14

**Authors:** Elena-Iuliana Flocea, Gabriela Mihalache, Bianca-Georgiana Anchidin, Ioana Gucianu, Marius-Mihai Ciobanu, Florina Stoica, Giulia Pascon, Daniel-Florin Lipșa, Paul-Corneliu Boișteanu

**Affiliations:** 1 “Ion Ionescu de la Brad” Iasi University of Life Sciences, 3 Mihail Sadoveanu Alley, 700490 Iasi, Romania; floceaiuliana5@gmail.com (E.-I.F.); bianca.anchidin@iuls.ro (B.-G.A.); ioana.gucianu@iuls.ro (I.G.); marius.ciobanu@iuls.ro (M.-M.C.); florina.stoica@iuls.ro (F.S.); paul.boisteanu@iuls.ro (P.-C.B.); 2 Integrated Centre of Environmental Science Studies in the North Eastern Region (CERNESIM), Department of Exact and Natural Sciences, Institute of Interdisciplinary Research, “Alexandru Ioan Cuza” University of Iasi, 11 Carol I, 700506 Iasi, Romania; gabriela.mihalache@uaic.ro; 3 Department of Agricultural, Food, Environmental and Animal Science, University of Udine, Sondrio 2, 33100 Udine, Italy; giulia.pascon@uniud.it

**Keywords:** fish, fish-based food product, orange peel, bioactive compounds

## Abstract

Fish-derived products are extensively acknowledged for their substantial role in fostering balanced diets and supporting a healthy way of life. This research is aimed at formulating, analyzing and evaluating a fish-based food product. The methodology adopted in this study adheres to contemporary food safety standards, prioritizing the utilization of minimal technological processes and natural ingredients, a focus that is gaining prominence within contemporary industrial practices. Thus, the proposal for a formulation obtained by integrating powders and extracts from plant byproducts (*Citrus*) represents a concrete application direction with real potential for commercialization. The product has been enriched with biocomponents derived from orange peel, namely orange extract (OE) and orange peel powder (PPO). The research focused on product development and the *in situ* evaluation of the effects of OE and PPO. The physicochemical composition, bioactive compound content, and antioxidant activity were evaluated, along with the microbiological status under post-opening refrigeration conditions, in order to simulate actual consumer use. In addition, the product’s color parameters and sensory attributes were analyzed. The results highlight significant potential for the development of a clean-label fish-based product, characterized by a simplified and easily implementable formulation, aligned with current production and consumption requirements. Compared to the control sample, both OE and PPO significantly influenced the analyzed parameters. Differences in physicochemical composition were observed in the experimental samples. In addition, PPO increased the antioxidant activity of the samples and the profile of bioactive compounds. Microbiological analysis, performed on day 0 and after 3 and 7 days of storage at 4 °C showed opening, confirmed the absence of *Escherichia coli* and *Staphylococcus aureus* in all samples and had an influence on the growth of fungi. The acceptability of fish-based products is often limited by odor perception, which is one of the main factors leading to consumer rejection. Sensory evaluation demonstrated that citrus-enriched samples were distinguished by the perception of particular sensory attributes. This formulation presents a practical solution to address this constraint, thereby enhancing the product’s sensory acceptability. The integration of OE and PPO yielded a more harmonized sensory profile, as evidenced by elevated hedonic scores and an intermediate placement in both principal component analysis (PCA) and external preference mapping. This research furnishes a thorough characterization of a fish-based food product, underscoring its potential as a viable option for balanced dietary regimens. Simultaneously, the findings support the product’s adherence to sustainability principles through the utilization of bioactive compounds sourced from plant byproducts, thus satisfying contemporary requirements for foods that possess an optimal nutritional profile and a diminished environmental footprint.

## 1. Introduction

Fish is recommended as a source of protein, a balanced mix of essential amino acids, healthy fats, vitamins, and minerals important for health. Fish consumption has been widely proven to have beneficial effects on health through clinical studies [1]. Functional foods or dietary supplements that contain bioactive molecules and have the ability to provide health benefits beyond their nutritional value are known as nutraceuticals. This term combines two words, nutrient and medicinal (pharmaceutical) component. In recent years, functional and bioactive compounds from natural sources, such as terrestrial and marine plants, animals, or even microorganisms, have become a sustainable solution that provides new molecules with strong biological activity [2]. Fish is a rich source of bioactive compounds, such as long-chain PUFAs (EPA and DHA), omega-3 PUFAs, peptides, protein hydrolysates, amino acids, minerals, vitamins, gelatin, collagen, fish oil, and fat-soluble vitamins, making it an important source of nutraceuticals and a beneficial dietary staple [3,4]. Population urbanization and health awareness among people with sedentary or stressful lifestyles are the main drivers of the global nutraceutical market growth [5]. Global public awareness of fish-based diets and their health/nutritional benefits is growing. The nutritional profile of fish includes bioactive compounds and other promising compounds with countless benefits for human health. Various reported research involving fish/marine-derived molecules reveals promising attributes, and the position of fish-derived nutrients as nutraceuticals continues to strengthen. These benefits have led several researchers to capitalize on these potentials in terms of incorporating and formulating functional foods and nutraceuticals [6].

One of the challenges of fish-based products is lipid oxidation. This is one of the main causes of deterioration in fish meat quality, due to its high content of polyunsaturated fatty acids. This oxidation is accelerated by catalysts such as hemoglobin and lipoxygenase, and common synthetic antioxidants (BHA, BHT, TBHQ, PG) are often ineffective in complex systems such as fish muscle. Plant substances rich in phenolic compounds have attracted attention because they can delay lipid oxidation through two main mechanisms: neutralizing free radicals and chelating pro-oxidant metal ions such as iron. Thus, plant polyphenols can effectively reduce oxidative reactions in fish by forming stable complexes with iron (Fe^2+^), which limits lipid degradation [7]. Consumer acceptance of natural food additives is higher than that of synthetic additives. The perception of naturalness is primarily linked to health. Natural products, such as phytochemicals derived from plants (phenols, essential oils, carotenoids, lignins, and other molecules), which have antioxidant and antimicrobial properties, offer numerous opportunities to combat protein degradation, lipid peroxidation, and also inhibit microbial growth, thereby improving the quality and shelf life of food products [8]. The holistic sensory experience creates a unified perception that influences consumer memory. Consumer interest in clean-label products highlights an accelerating trend toward products without artificial additives. From a sensory perspective, food appeal is significantly influenced by how additives actively participate in the organoleptic properties of the final product. The brain simultaneously processes multimodal integrated stimuli from organoleptic properties, reaching the orbitofrontal cortex and other regions involved in the neuroprocessing of the final product. The reformulation and development of meat products requires a detailed analysis of the impact of additives on sensory properties, contributing to the shaping of consumption trends [9,10]. Martínez et al., 2019 [11] found that natural extracts from pomegranate, rosemary, and olive (rich in phenolic compounds like hydroxytyrosol) can act as effective antioxidants and antimicrobials, replacing synthetic additives. They have demonstrated strong activity in vitro (through DPPH, ABTS, FRAP, ORAC, and disc diffusion tests) and in vivo in fish products, where they delayed lipid oxidation and microbial growth (TVC, TCC, *E. coli*, *L. monocytogenes*). Their use in fish cakes extended shelf life and reduced sensory deterioration, highlighting the potential of natural extracts as preservatives in the food industry [12]. Ethanol extracts from bitter orange albedo showed significant antioxidant activity in fish lipids, reducing lipid oxidation (peroxide value, para-anisidine, and TBARS) during storage. Concentrations of 1.0 mg g^−1^ and 2.0 mg g^−1^ provided the best results, maintaining lipid quality, while higher concentrations (5.0 and 10.0 mg g^−1^) had negative effects on oxidation stability. Thus, choosing the optimal extract concentration is essential for preventing lipid oxidation [13].

According to the circular economy, the utilization of *Citrus* waste as a renewable biological resource is becoming increasingly important for the citrus processing industry to reduce harmful environmental impacts and recycle bioactive compounds. *Citrus* peel powder can be used as a functional food ingredient to extend shelf life and provide health benefits. *Citrus* peel waste is rich in extractable and non-extractable phenols, along with dietary fiber and antioxidants, which confer numerous health benefits, including antioxidant, antimicrobial, anti-inflammatory, and cardiometabolic effects. Non-extractable phenols, in particular, have sustained release and colonic metabolism that enhance bioactivity and modulate the gut microbiota. Despite promising in vitro and some in vivo data, there are limitations to in situ studies [14].

In the context of current trends in food industry and science, this study aims to develop, characterize and validate a product based on fish fillets (*Cyprinus carpio*), enriched with plant-based biocomponents obtained from orange peel powder and liquid extract. The aim of the research is to assess in situ the effects of these components on the physicochemical, microbiological and sensory properties of the finished product, as well as on the antioxidant capacity and the content of bioactive compounds. The study also addresses the use of minimal technological processes and components of natural origin, in parallel with the use of agri-food byproducts as sources of bioactive compounds with technological potential in fish-based products.

## 2. Materials and Methods

### 2.1. Materials

The type of fish used (*Cyprinus carpio*) was purchased from the local market and certified for quality in accordance with EU regulations on food safety and traceability of animal products, including Regulation (EC) No. 854/2004 [15] and Regulation (EU) No. 1169/2011 [16]. The liquid extract from orange peel (EO) was purchased from a local pharmacy, and the orange peel powder (PPO) was purchased from a local manufacturer with quality certifications. To avoid conflicts of interest, the brand names of the manufacturers were kept anonymous. These experimental plant-based biocomponents were chosen for use in the study to analyze a scenario similar to the real one, in accordance with the relevant regulations, Regulation (EC) No. 178/2002 [17] on food safety, Regulation (EU) No. 1169/2011 [16] on consumer information, and other regulations applied in the field of food and natural product safety.

### 2.2. Sample Preparation

**Figure 1:**
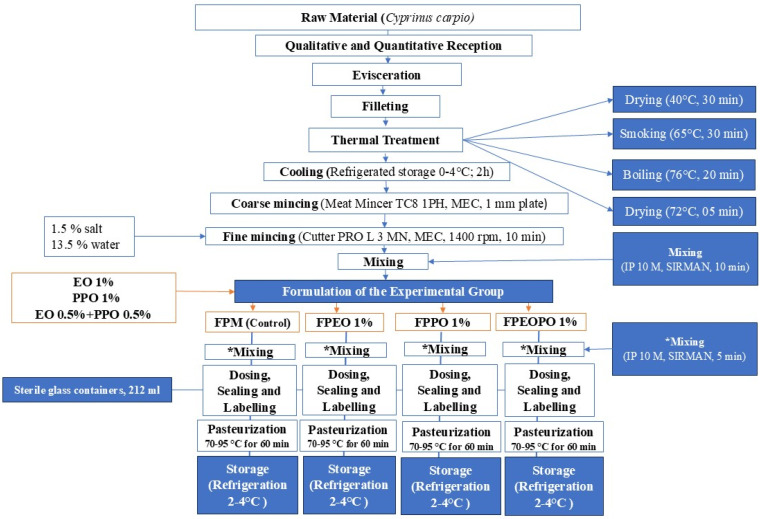
The technological flow of obtaining experimental samples; * The samples were mixed separately using the same equipment.

The process flow for obtaining experimental samples was designed to ensure a reproducible, controlled process adapted to the objectives of the study. Figure 1 illustrates all stages of the technological workflow, from the receipt of raw materials and careful monitoring of the process to the production of the final product and ensuring food safety conditions in accordance with the ISO 22000 standard [18]. Raw material processing was carried out under controlled sanitary conditions, with monitoring of critical parameters (temperature, time) to prevent the degradation of sensitive components and maintain stability. A qualitative and quantitative inspection was performed; subsequently, the fish underwent gutting and filleting operations. The filleted samples were placed in an Automatic Smoking Cell KWG1E (STAWIANY, Pszczółki, Poland), where specific heat treatments were applied.

Heat-treated fish fillets formed the basis of the samples studied. The heat treatments are presented in Table 1.

After completing the heat treatment described in Table 1, the fish fillets (*Cyprinus carpio*) were transferred to a refrigeration room for the cooling stage, where they were held at temperatures ranging from 0 to 4 °C for 2 h. The preparation and processing of the experimental samples were carried out in a processing area with a controlled temperature of 10–20 °C, in accordance with the requirements applicable to fish and fish product processing facilities. The fillet was coarsely minced using a Meat Mincer TC8 1PH, MEC (MEC EUROPE SRL, Rimini, Italy), equipped with a 1 mm perforated screen. The resulting material was then finely minced using a PRO L 3 MN, MEC mincer (MEC EUROPE SRL, Rimini, Italy) for 10 min at a blade speed of 1400 rpm, in the presence of 1.5% salt and 13.5% water. The mixture was homogenized using SIRMAN IP 10 M (SIRMAN, Curtarolo, Italy) for 10 min, until a uniform and homogeneous consistency was achieved. The resulting mixture was weighed and divided into equal portions for the formulation of the experimental variants and the incorporation of the components used in the study, as shown in Table 2.

Experimental samples of spreadable paste were developed, to which 1% EO and 1% PPO were added, and the last batch consisted of 0.5% EO + 0.5% PPO relative to 400 g of heat-treated fish fillet. The control sample included a spreadable paste made from fish fillets (*Cyprinus carpio*), obtained by applying the same technological flow as the experimental samples, but without the addition of plant-based biocomponents, being used as a reference system. The sample with orange peel liquid extract (1%) included a spreadable paste made from fish fillets (*Cyprinus carpio*), to which orange peel liquid extract was added, applying the same technological flow as in the control sample, to allow for a comparative evaluation of the effect of the plant biocomponent on the quality of the final product. The sample with orange peel powder (1%) included a spreadable paste made from fish fillets (*Cyprinus carpio*), supplemented with powder obtained from orange peel. The sample with liquid extract and orange peel powder (0.5% + 0.5%) was formulated by incorporating the liquid extract from orange peels and the corresponding powder into the protein matrix of the spreadable paste made from fish fillets, in order to evaluate the synergistic effect on the stability and quality of the final product. The percentages were established based on preliminary tests conducted before the main experiments. Each sample was homogenized using the SIRMAN IP 10 M for 5 min until a uniform and homogeneous consistency was achieved. No additional spice mixtures or aromatic additives were included in the product formulation, as the experimental approach was geared toward developing a simplified food product that would allow for the precise evaluation of the influence of bioactive extracts on the physicochemical, microbiological, and sensory characteristics of the final product. At the same time, this reformulation strategy aimed to create a clean-label product, characterized by a reduced number of ingredients and a production process adapted to responsible consumption. The filling process was performed automatically into sterile, recyclable glass containers (212 mL) using a REX RVF 327 filling machine (REX-Technologie GmbH & Co. KG, Thalgau, Austria) equipped with a 14 mm filling head. After filling and hermetic sealing, the samples underwent pasteurization at 70–95 °C for 60 min and were subsequently stored under refrigerated conditions at 2–4 °C.

### 2.3. Physicochemical Analysis

The samples were analyzed in triplicate for their proximate composition (moisture, protein, lipid and ash) according to AOAC standard methodology [19]. Dry matter (DM) was determined by drying the samples in an oven at 105 °C for 4 h before weighing (930.15). Ashes were determined by incineration of the samples in a muffle at 550 °C for 4 h (942.05). Crude protein (CP, N × 6.25) was measured according to Kjeldahl method (976.05). Lipid fraction was extracted following Folch et al. (1957) [20].

### 2.4. Antioxidant Activity and Bioactive Compounds

#### 2.4.1. Extraction of Phytochemical Compounds

The extraction of bioactive compounds from plant-based powders was performed using ultrasound, using the method described by Gavril (Rațu) et al. (2024) [21], with slight modifications. In short, 1.0 g of powder was combined with 10 mL of solvent mixture n-hexane/acetone (3:1, *v*/*v*) or 10 mL of 70% ethanol (only for the extraction of total polyphenols and total flavonoids, antioxidant activity by the DPPH method) and underwent an ultrasonic treatment for 40 min at 35 ± 2 °C, respectively, and the frequency was 40 kHz. After recovering the resulting extract, it was then centrifuged for 10 min at 6000 rpm and 10 °C. The supernatant was collected after separation and characterized phytochemically.

#### 2.4.2. Phytochemical Characterization of Extracts

##### Determination of the Total Carotenoid Content

Extracts obtained in the extraction solvent (hexane: acetone, 3:1) were read at = 450 nm for total carotenoids, = 470 nm for = carotene and = 503 nm for lycopene [22]. Carotenoid, ß-carotene and lycopene concentrations were calculated using Equation (1):

(1) $$\mathrm{Total}\;\mathrm{carotenoid}\;\mathrm{content}\;(µg\;g^{-1}\;\mathrm{dry}\;\mathrm{matter})/ß-\mathrm{carotene}/\mathrm{lycopene}\;=\;\frac{A\times Mw\times Df)}{m\times L\times Ma}$$

where

A—absorbance of the sample to be analyzed at wavelengths of = 450, 470 and 503 nm;

Mw—molecular weight;

Df—sample dilution factor;

m—mass or weight extract;

L—length of the optical path of the bowl (1 cm);

Ma—molar absorption, for total carotenoids (2500 L mol^−1^ cm^−1^), ß-caroten (2592 L mol^−1^ cm^−1^), licopen (3450 L mol^−1^ cm^−1^).

##### Determination of the Total Flavonoid Content

The colorimetric method described by Turturica et al., 2016 [23] was used to determine the total flavonoid content. Over 0.25 mL diluted plant-based powder extract, 1.25 mL distilled water and 0.075 mL sodium nitrite solution 5% were added. The mixture was left at room temperature for 5 min, after which 0.15 mL 10% aluminum chloride solution was added. After a 6 min rest, 0.5 mL of sodium hydroxide 1M and distilled water up to a volume of 3 mL were added. The absorbance of the mixture was measured immediately at 510 nm. The total content of flavonoids is determined by means of the standard catechin curve, which is expressed in mg catechin equivalents/g dry matter.

##### Determination of Total Polyphenol Content

The total polyphenol content was determined by the Folin–Ciocalteu colorimetric method described by Turturica et al. (2016) [23]. The method is based on the chemical reduction of Folin–Ciocalteu reagent, a mixture of tungsten and molybdenum oxides. Briefly, 200 μL of extract was diluted in distilled water (15.8 mL), over which 1 mL of Folin–Ciocalteu reagent was added. After 10′, 3 mL of 20% sodium carbonate solution was added, and after another 60′, during which the mixture was kept in the dark, the absorbance at the wavelength of 765 nm was determined. The content of phenolic compounds was expressed as mg gallic acid/g dry matter, using a standard gallic acid curve.

#### 2.4.3. Determination of Antioxidant Activity

##### Method of the Radical DPPH (2,2-Diphenyl-1-Pichrylylhydrazil)

To determine the antioxidant activity, the DPPH method (2,2-diphenyl-1-picrylhydrazyl) described by Turturica et al. 2016 [23] was used. In short, a volume of 3.9 mL of DPPH solution reacts with 100 μL of plant-based extract for 60 min, at room temperature, in the dark. The absorbency of the solution was read at 515 nm. The antioxidant activity of the extracts was expressed in μmol Trolox/g dry matter by reference to a standard curve [24]. The percentage of inhibition of the DPPH radical was assessed against the sample using Equation (2):

(2) $$\mathrm{DPPH}\;\mathrm{Inhibition}\;(\%)\;=\;\;\frac{A-B}{A}\;\times 100$$

where

A—Absorption read for sample control;

B—Absorption read for the sample analyzed.

### 2.5. Colorimetric Analysis

The color characteristics of the samples were assessed using a Chroma Meter MINOLTA, CR-410 model (Konica Minolta, Osaka, Japan), based on the CIE Lab scale. The results of the color measurements were expressed in terms of parameters L*, a* and b*, where L* represents brightness (with values from 0 for black to 100 for white), a* indicates the degree of red (−a* for green, +a* for red), and b* reflects the degree of yellow (+b*) or blue (−b*). After calibrating the equipment on a white plate, the CIELAB parameter values from three samples were collected. The angle of nuance was also calculated using the formula Hue angle = 180 + arctan (b*/a*) for dial II (−a*, +b*), thus describing the visual appearance of the color [25]. Chroma, which represents the intensity of color, was determined according to Equation (3):

(3) $$\left(\sqrt{{\left(a^{*}\right)}^{2}+{\left(b^{*}\right)}^{2}}\right)$$

and the total color difference (ΔE) was calculated according to Equation (4):

(4) $$\Delta E\;=\;(\sqrt{{L^{*}}^{2}+{a^{*}}^{2}+{b^{*}}^{2}}$$

### 2.6. Microbiological Analysis

The microbiological analysis of the samples was done according to Regulation (EC) No 2073/2005 [26] for fish products. The growth of *E.coli* and *S. aureus* was monitored immediately after processing the fish paste (day 0), until 7 days of storage at 4 °C. Briefly, 1 g of sample was homogenized with 9 mL of buffered peptone water and serial dilutions (10^−1^, 10^−2^) were done. A volume of 1 mL from each dilution was inoculated on plates with selective chromogenic agars, Rapid *E. coli* 2 and Rapid *Staph* (Bio-Rad, Marnes-la-Coquette, France), and incubated for 24 h at 37 °C [27]. In addition, samples were also cultured on Dichloran Rose Bengal Chloramphenicol (DRBC) medium, to evaluate the development of molds, and incubated at 25 °C for 5 days [28]. After incubation, the presence or absence of *E.coli* and *S. aureus* was registered, and the molds were enumerated and expressed as logarithmic values of colony-forming units per gram of product (log CFU·g^−1^). It is important to point out that the 7-day interval investigated in this study does not reflect the shelf life of the product in the sealed state, but the microbiological safety period after opening, under refrigeration conditions. This approach was chosen to simulate actual consumer conditions, in which the product becomes perishable after disposal and is used within a short period of time, thus allowing to assess the practical relevance of microbiological stability and the efficiency of the natural components used.

### 2.7. Sensorial Analysis

#### 2.7.1. Consumer Sensory Evaluation

The sensory assessors were selected, trained, and monitored in accordance with ISO 8586 [22]. The sensory panel consisted of 24 semi-trained panelists, aged between 20 and 42 years. Sensory evaluations were conducted in a standardized test room, in compliance with the requirements of ISO 8589 (ISO, 2007) [22]. Participants were selected among healthy, non-smoking individuals who were not under medical treatment and did not suffer from chronic conditions such as hypertension or cardiovascular diseases. All participants were instructed not to have experienced cold symptoms or other conditions that could affect sensory perception for at least one week prior to the sensory evaluation and to have previous experience in the sensory evaluation of meat products. The training program of the sensory panel included the identification and description of aromas specific to meat products, as well as familiarization with the procedures for using the response scale. Sensory evaluations were carried out only after the panel demonstrated an adequate level of repeatability and consistency in sample assessment. The sensory evaluation of the products was performed over three distinct tasting sessions. Samples were coded with three-digit numerical codes and presented to the panelists in a randomized order, in order to reduce potential influences on sensory evaluation. Between samples, panelists were instructed to rinse their oral cavity with still water, to ensure taste neutralization and to minimize carry-over effects from the previous sample.

##### Hedonic Sensory Evaluation of the Products

Considering that the consumer hedonic tests in this study were conducted under controlled conditions, the provisions of ISO 11136 [29] were complied with throughout the hedonic sensory evaluation. The acceptability of the analyzed fish products was assessed using a 9-point hedonic scale (1 = dislike extremely, 2 = dislike very much, 3 = dislike, 4 = dislike slightly, 5 = neither like nor dislike, 6 = like slightly, 7 = like, 8 = like very much, 9 = like extremely). The mean hedonic scores obtained for each sensory category (appearance, aroma, taste, texture, and overall liking) were graphically represented using a radar chart in order to facilitate the comparison of the hedonic profiles of the analyzed samples.

##### Check-All-That-Apply (CATA)

The Check-All-That-Apply (CATA) method was used to describe the sensory profile of the products based on the selection of sensory terms considered relevant by the evaluators. The relevance of extended lists of terms is related to the idiosyncratic nature of consumer perception and the way sensory sensations are expressed, as well as to the ability of this method to capture subtle similarities and differences among the analyzed samples [30]. The use of the CATA method contributes to reducing potential sources of bias in consumer-based sensory profiling studies [31]. In the present study, the CATA terms were established by the members of the sensory panel (Table 3), based on their sensory perceptions expressed during the familiarization stage with the products. This approach was adopted in order to minimize effects related to attribute order and individual interpretation variability, thus ensuring a more accurate description of the sensory differences among samples. Based on the selection frequencies of the CATA terms, occurrence matrices were constructed, and Cochran’s Q test was applied to identify the sensory attributes that significantly discriminated among the analyzed samples (*p* < 0.05).

##### Quantitative Descriptive Analysis (QDA)

Quantitative Descriptive Analysis (QDA) was conducted using a set of sensory attributes established by the authors of the study and defined prior to evaluation (Table 4). The semi-trained sensory panel assessed the intensity of each sensory attribute using a 9-point numerical scale, where the scores reflected the perceived intensity level rather than hedonic preference, as is the case in consumer hedonic tests.

For each sensory attribute and sample, means and standard deviations were calculated. Subsequently, a two-way analysis of variance (ANOVA) was applied to evaluate the effects of the sample, sensory attributes, and their interaction. Due to the presence of a significant interaction effect, a one-way ANOVA was further performed separately for each sensory attribute.

##### Principal Component Analysis (PCA)

Principal component analysis (PCA) is a widely used multivariate statistical method [32] and was applied to the data obtained from the Quantitative Descriptive Analysis (QDA) (Table 4) in order to highlight the relationships between sensory attributes and fish paste samples, as well as to discriminate among the sensory profiles of the analyzed products.

##### External Preference Mapping (PrefMap)

Internal and external preference mapping are widely used tools for product optimization (Worch, 2013) [33]. External preference mapping (PrefMap) was performed using the QDA values of the 18 sensory attributes together with hedonic scores, allowing for the grouping of panelists into preference clusters and the positioning of samples in the PrefMap space to describe overall consumer preferences.

### 2.8. Statistical Analysis

All tests were performed in triplicate (n = 3), and the results are expressed as mean ± standard deviation. The data was analyzed using unidirectional variation analysis (ANOVA), followed by Tukey’s HSD post hoc test, to test the differences between the groups. The differences were considered statistically significant, *p* < 0.05. Statistical analyses related to sensory evaluation were performed using XLSTAT (version 2025.27.1.2) and SPSS Statistics, version 26.

## 3. Results

### 3.1. Physicochemical Analysis

Experimental samples from fish fillets enriched with plant biocomponents have a balanced nutritional value. According to Table 5, the chemical composition was influenced by the synergy between biocomponents in the food matrix based on fish fillets.

The total protein content was highest in the experimental sample with 0.5% EO + 0.5% PPO (17.05 ± 0.48%), being significantly different (*p* < 0.05) from the blank sample. These differences can be attributed to bioingredient-induced changes in the food matrix, in particular on the distribution of components and moisture content, which influence the percentage values expressed [34]. By contrast, the lowest protein content (15.03 ± 0.83%) was recorded in the sample with 1% PPO; the difference from the blank sample was also statistically significant (*p* < 0.05). Lipids were significantly (*p* < 0.05) influenced by the introduction of plant biocompounds. A decrease in the experimental samples was observed, with the lowest amount recorded in the experimental sample by 0.5% EO + 0.5% PPO (6.87 ± 0.11%), a significant difference (*p* < 0.05) from the control sample (8.1 ± 0.25%). Polyphenols can interact with lipids and alter fluidity, with permeability being harder to extract [35]. In the case of ash content, the differences were not significantly different between the samples, but a slight increase was observed in samples with orange peel powder and orange peel extract. Polyphenols can bind with Fe and other metal ions (Cu, Zn) by phenolic groups, forming stable complexes [36]. The incorporation of orange peel powder (PPO) and orange peel liquid extract (EO) causes an increase in dry matter content. Plant-based powders may be characterized by relatively high dry matter content, primarily due to their low moisture level and the presence of structural carbohydrates (e.g., dietary fiber) [37]. However, due to the complexity of the food matrix, in the case of the FPPO sample, the percentage of dry matter is the lowest of the analyzed samples. *Citrus* peel powders have significant mineral content, which contributes to the growth of dry matter and can alter the mineral profile of the product [38]. The moisture content of the product is high, which gives the product greater microbiological problems. In experimental samples, statistical differences are observed compared to the blank sample (*p* < 0.05). The addition of powdered and extract plant biocomponents can retain water by increasing the solids content and reducing the exudate when processing [39,40]. The biocomponents used, namely orange peel powder and orange peel extract, resulted in significant changes in the chemical composition of the product, according to the experimental results obtained. Their integration into the formulation of fish products can be considered an effective nutritional strategy, contributing to the development of products with improved nutritional value and potentially beneficial on food diets based on fish products. The variations observed between samples can be attributed to the complexity of interactions within the food matrix, which influence the distribution of components and compositional reporting.

### 3.2. Antioxidant Activity and Bioactive Compounds

Orange peel contains several classes of bioactive compounds that have been quantified in recent studies [41,42]. Quantitative values and identifiers of compounds reported in the literature indicate high levels of flavonoids and carotenoids. Table 6 shows the content of bioactive compounds and antioxidant activity in the orange peel powder and liquid extract from orange peel used in the study.

Quantitative values reported in the literature indicate high levels of flavonoids, carotenoids, and orange peel polyphenols. Hesperidine was quantified at 16.26 mg/g of dry weight in optimized extracts, and other reported dominant flavonoids include naringin, hesperetin, naringenin, and narirutin [34,43]. For the analyses of bioactive compounds and antioxidant activity, an extract obtained from the biocomponents used, prepared according to the described extraction method, was used. Regarding the characterization of bioactive compounds in our study, the quantity of total flavonoids in the powder (OP) is lower compared to that of the orange peel extract (EO). The extract from orange peel powder (PPO) still has a higher quantity of polyphenols (3.88 ± 0.121 mg GAE/100 g dw) compared to the liquid extract from orange peel (EO) (1.23 ± 0.018 mg GAE/100 g dw). The extracts reported a total polyphenol content of up to 34.71 mg GAE/g dry weight in optimized procedures and ~80 mg catechol equivalents/100 g dry weight for sweet orange extracts using ethanol extraction [44,45].

In our case, orange peel powder (PPO) reported a much higher total polyphenols value (12.78 ± 0.01 mg GAE/100 g dw) compared to orange peel extract (EO), which has a value of 8.53 ± 0.12 mg GAE/100 g dw. Similar to the total carotenoid count, PPO shows a higher quantity (2.06 ± 0.000 mg GAE/100 g dw), while EO shows a lower quantity (1.44 ± 0.01 mg GAE/100 g dw). The antioxidant activity of EO and PPO is high by the DPPH method, TE/g dw. According to Camacho M. del M. et al., 2022 [46], orange peel gave ~1.67 g GAE/100 g TPC and inhibition ~59% DPPH under reference conditions for water bath extraction. Multiple studies report strong in vitro antioxidant and antimicrobial effects for shell extracts applied in food matrices and against pathogens [43,46].

The phytochemical characterization and antioxidant activity of orange peel powder (PPO) and orange peel extract (EO), as well as their impact on experimental samples from fish fillets (*Cyprinus carpio*), are shown in Table 7. Phytochemical analysis highlights the presence of amounts of bioactive compounds, especially total polyphenols and flavonoids, in both PPO and EO, confirming their potential as natural sources of antioxidants. The observed differences between the two forms of orange peel valorization can be attributed to the applied technological processes, which influence the degree of extraction and bioavailability of phytochemical compounds. The antioxidant activity assessed by the DPPH method indicates an antioxidant capacity for both plant biocomponents, with variations depending on the application form and the food matrix. The incorporation of PPO and EO into experimental samples of fish fillets led to a significant increase in antioxidant capacity compared to the blank sample. This behavior is relevant in the context of the high susceptibility of fish to oxidative processes.

For the FPPO sample, the total flavonoid quantity is highest (1.025 ± 0.030 mg CE/100 g dw) compared to the other samples. PPO showed stability of bioactive compounds following pasteurization treatment of 70–95 °C for 60′. The lowest amount of total flavonoids among the batches of the experiments is presented in the FPEO sample with orange peel extract (0.604 ± 0.00 mg CE/100 g dw). The situation of total polyphenols is similar to that of total flavonoids; the PFPPO has a quantity of 1.31 ± 0.02 mg CE/100 g dw, being significantly increased (*p* < 0.05) compared to the PPEO sample, where the quantity of polyphenols is 0.93 ± 0.01 CE/100 g dw. Studies on food-type spreads from spreadable fish are limited, but in Shoja’s study 2024 [47], the use of orange peel extract in chitosan coating to preserve fish fillets shows antioxidant activity and extended shelf life. The antioxidant activity by the DPPH method (*p* < 0.05) of TE/g dw is significantly increased in the FPPO sample with orange peel powder 12.14 ± 0.17 mg CE/100 g dw) than with orange peel extract (7.60 ± 0.14 mg CE/100 g dw). But also, the FPEOPO sample that has the mixture of EO and PPO can be a promising formulation for antioxidant activity, showing values of 10.28 ± 0.12 mg CE/100 g dw. According to the study by Mei-Ling Chen et al. (2011) [48], drying orange peels at high temperatures (70–100 °C) does not necessarily lead to the degradation of phenolic compounds. On the contrary, extracts obtained from dried peel at 100 °C showed significantly higher polyphenols and flavonoids content as well as superior antioxidant capacity as assessed by DPPH, ABTS and Fe^2+^ ion chelation tests compared to extracts from dried peel at lower temperatures. These results suggest a relatively high thermal stability of the antioxidant compounds in the orange peel. In addition, increased stability can be attributed, at least in part, to the predominance of glycosylated flavonoids, which exhibit superior thermal resistance to aglyconic forms [49].

### 3.3. Colorimetric Analysis

The color of the product is a determining factor in the visual evaluation, significantly influencing the consumer’s perception of appearance, being closely correlated with the presence and concentration of the biocomponents introduced in the product formulation. Food neophobia is a psychological feature of the consumer defined by the consumer’s technique of rejecting unknown foods or products obtained by new technologies, perceived as atypical. In this context, color parameters are an essential indicator in the characterization of a new food, since they significantly contribute to the formation of the first visual impression and the process of familiarization of the consumer with the product. A true chromatic appearance, correlated with natural components and applied technology, can reduce initial reluctance, facilitating sensory acceptability and forming a safety perception of product quality [50]. According to Table 6, the color parameters of the experimental samples were expressed in L* (D65), a* (D65), b* (D65), C* (Chroma) H* (Hue Angle), ∆E. The results indicate that the plant biocomponents used in the experimental samples influenced the parameters compared to the blank sample.

An increase in the parameter L* (D65) is observed for significantly different PPEO and PFEOPO samples (*p* < 0.05) with the MMF sample. The incorporation of the PPO mainly gave the experimental samples a lighter color and a more pleasant appearance. In the case of the parameter b* (D65), we observe significant differences (*p* < 0.05). This demonstrates the ability to color due to the carotenoid pigments present. In Mahmoud M. H et al., 2017 [51], the addition of orange peel powder/extracts tends to increase brightness (L*) and (b*), while redness (a*) decreases at certain levels, meaning the product becomes lighter and slightly yellowish compared to the control. This observation is reported in the analysis of the quality of burgers and in studies on minced meat. The effect on color depends a lot on the level of incorporation, the product matrix (meat vs. baked product vs. emulsion), the processing temperature (baking, pasteurization can intensify or degrade pigments) and the presence of fats (which can accentuate yellow) [52]. The value of the hue angle measures the chromaticity or tone of the color, while the color value represents the color saturation. Chroma shows a trend of an increase in experimental samples compared to the witness sample. According to Table 8, we see a significant increase (*p* < 0.05) compared to the other samples of the parameter ∆E, which demonstrates the much greater impact of PPO than EO.

### 3.4. Microbiological Analysis

Table 9 presents the results of the microbiological tests of fish paste samples supplemented with orange peel extract, orange peel powder or their mixture, as well as the unsupplemented control. The samples were evaluated for the presence of *E. coli*, *S. aureus* and molds at the end of the processing (day 0), and after 3 and 7 days of storage at 4 °C after undoing. The results showed that regardless the storage day and the sample analyzed neither *E. coli* nor *S. aureus* were detected, suggesting the effectiveness of the pasteurization process and the hygienic conditions of production. Regarding mold presence, the results varied depending on the sample. For instance, the samples that were not supplemented (FPM) and the fish paste containing orange peel powder (FPPO) showed an increase in the molds counted during the 7 days of storage. In the samples supplemented with orange peel extract (FPEO), molds were registered only at day 0 (1 log CFU·g^−1^), while after 3 and 7 days of storage, no mold was detected. When the mixture of orange peel extract and powder (FPEOPO) was used, molds were registered only after 7 days of storage. These results can indicate that the presence of orange peel extract alone or in combination with orange peel powder in the fish paste could have anti-mold effects. Different studies have reported the antifungal properties of orange peel. Among the species of fungi on which orange peel exerted inhibitory activities are *Aspergillus niger*, *Aspergillus flavus*, *Rhizopus* spp., *Mucor* spp., *Penicillium* spp., *Fusarium oxysporum*, *Geotrichum candidum*, and *Pythium* spp. The presence of polyphenols and flavonoids were especially linked with the antifungal activity [53,54,55,56,57,58,59]. However, the rate of inhibition depends on many factors such as the type of polyphenol or flavonoid, their concentration, or the fungal species. For example, in a study done by Hernández et al., 2019 [53], the flavonoids present in the orange peel extract had limited effects on the growth of *Monilinia fructicola*, *Botrytis cinerea* and *Alternaria alternata*, while ferulic and p-coumaric acids showed high rates of inhibition. Moreover, when flavonoids were mixed with phenolic acids, their activities were not synergic [53]. On the other hand, in other studies, flavonoids such as hesperidin, hesperitin glucoside, hesperitin glucoside laurate, limonene or citral showed inhibitory effects against different fungi like *Fusarium semitectum*, *Penicillium expansum*, *Aspergillus parasiticus*, *Aspergillus flavus*, *Fusarium verticillioides*, *Aspergillus niger*, *Penicillium italicum* or *Rhizopus* spp. [59]. In line with these observations, our results indicate that differences in mold growth among the formulations were not directly related to the total flavonoid and polyphenol content. Although the highest concentrations of flavonoids and polyphenols were detected in fish paste supplemented with orange peel powder, mold growth was still observed. This indicates that factors such as compound form, distribution, and interactions with the food matrix may play a role in mold development during storage.

### 3.5. Consumer Sensory Evaluation

An essential part of food product development and the introduction of new products to the market involves the evaluation of consumer liking [60]. Since it is difficult to fully understand consumer perception, research on acceptability and preferences is generally aimed at determining how the intensity of perceptible characteristics relates to the level of product acceptance [61,62].

#### 3.5.1. Hedonic Evaluation of the Analyzed Products

Hedonic sensory evaluation is used to assess the degree of consumer liking and represents an essential component of sensory analysis. Its importance lies in the fact that a product may exhibit individually positive sensory attributes, yet these attributes do not always combine to generate a coherent and pleasant overall sensory experience [63]. In the present study, hedonic evaluation was performed using a 9-point hedonic scale, and the main global sensory parameters evaluated were appearance, aroma, taste, texture, and overall liking. Since its introduction, the 9-point hedonic scale has been the most widely applied method for assessing consumer preferences and product acceptability in food studies [64]. The widespread acceptance of the 9-point hedonic scale is primarily due to its clear categorical structure and reduced number of response options compared to other scaling methods, such as magnitude estimation, making it easier to use for both participants and researchers. Moreover, its simple design allows for efficient application among a wide range of assessors without requiring specialized training [65].

**Figure 2:**
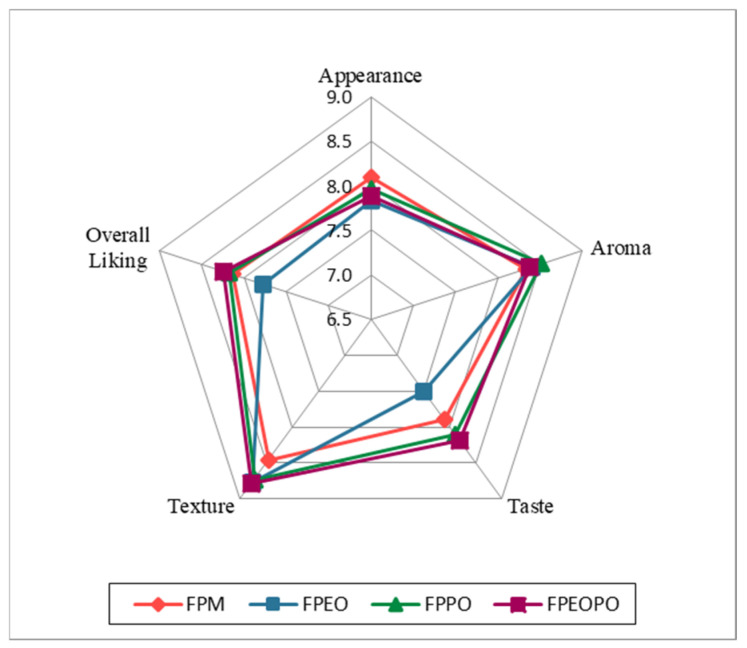
Radar plot illustrating the sensory profile (appearance, aroma, taste, texture and overall liking). FPM—control spreadable fish paste without orange-derived ingredients; FPEO—spreadable fish paste formulated with orange extract; FPPO—spreadable fish paste formulated with orange peel; FPEOPO—spreadable fish paste formulated with a combination of orange extract and orange peel. Values represent mean sensory scores obtained using a 9-point hedonic scale, where higher values indicate greater sensory acceptance.

The radar chart (Figure 2) graphically illustrates consumers’ hedonic perceptions regarding appearance, aroma, taste, texture, and overall liking for the analyzed fish paste samples. The sensory attributes, appearance and aroma exhibited similar values across all samples, indicating that the addition of orange extract and/or orange peel did not induce negative visual or olfactory changes. These findings are consistent with those reported by García et al. (2007) [66] for low-fat emulsified sausages supplemented with dietary fibers (apple, peach, and orange), where orange fiber obtained the most favorable sensory scores and was considered the most suitable option for preserving sensory quality in meat products. In contrast, taste was the sensory attribute that showed the greatest variability among samples. Fish paste formulated with orange extract alone (FPEO) received the lowest taste scores, suggesting a negative influence of this additive on gustatory perception. However, when orange extract was combined with orange peel (FPEOPO), the resulting product exhibited a superior taste profile compared to the other samples, with the exception of FPPO (fish paste with orange peel), whose scores were very close, ranging between 8 and 8.5, values approaching the maximum possible score of 9.

The texture attribute recorded the highest scores among all evaluated hedonic characteristics, highlighting that texture was perceived as optimal for a paste-type product. Furthermore, the addition of orange extract and orange peel did not negatively affect this parameter; on the contrary, it led to an improvement. Fish paste samples containing orange-derived ingredients (FPEO, FPPO, and FPEOPO) obtained higher texture scores than the control sample (FPM), indicating a higher consumer preference and an enhanced textural perception. Regarding overall liking, a highly favorable general acceptability was observed for samples containing orange peel (FPPO) and for those formulated with a combination of orange extract and orange peel powder (FPEOPO), whose mean values were slightly higher than those of the control sample (FPM). Among all samples, FPEOPO achieved the highest overall liking score (≈8.30), suggesting that the combination of orange peel and orange extract resulted in a sensory profile particularly appreciated by consumers and slightly superior to that of the control fish paste. Nevertheless, these three samples showed a high degree of similarity in terms of overall hedonic perception, with no major differences detected. The only sample that consistently diverged from this trend was FPEO, which exhibited the lowest overall liking score, indicating that the exclusive use of orange extract exerted a mildly unfavorable effect on global acceptability.

Overall, the hedonic results demonstrate that solid plant-based additives, such as orange peel powder, confer greater sensory benefits to fish-based meat products compared to liquid plant-based additives, which appear to be less well tolerated in this type of formulation. The sensory superiority of samples containing orange peel powder (FPPO and FPEOPO) observed in this study is supported by previous research on processed meat products, where orange peel powder led to sensory scores comparable to or higher than those of control samples. Mahmoud et al. (2017) [51] reported that beef burgers containing 5% orange peel achieved high overall sensory scores, although slightly lower than those of the control sample, demonstrating good consumer acceptance across concentrations ranging from 2.5 to 10%. Similarly, Ibrahim et al. (2018) [67] showed that beef patties supplemented with 2% orange peel obtained higher global sensory scores than the control, reinforcing the favorable sensory impact of orange peel addition.

Although the literature includes numerous studies on the use of orange extracts in meat and fish products, most of these investigations focus primarily on improving shelf life, oxidative stability, and microbiological quality rather than on their incorporation into ready-to-consume processed products. For example, Haque et al. (2020) [68] evaluated the effects of orange peel extract (0.2–0.4%) on meat quality during 60 days of frozen storage, reporting preservation of raw material quality. Likewise, Murshed et al. (2023) [69] examined the influence of orange peel extract on the quality and shelf life of chevon over 15 days, with results discussed mainly from a preservation standpoint. Consequently, available data regarding the sensory impact of such extracts in processed products remain limited, justifying the need for applied sensory evaluation in fish paste formulations containing orange extract. The predominant use of orange extract instead of orange peel in these studies can be explained by its ease of uniform application and efficient diffusion of bioactive compounds, which is essential for research focused on preservation and stability rather than sensory performance.

#### 3.5.2. Check-All-That-Apply (CATA)

The results of the CATA analysis (Table 10 and Figure 2) provide a qualitative description of the sensory profile of the fish paste samples by highlighting both the sample-specific sensory attributes and the differences observed among samples. Participants were presented with a predefined list of sensory descriptors together with the fish paste samples (Table 3), and the assessors were instructed to select all attributes they considered applicable to each sample.

Table 10 presents the selection frequencies of the sensory attributes evaluated using the CATA method (23 attributes), along with the results of Cochran’s Q test, which was applied to identify significant differences among fish paste samples (*p* < 0.05). The selection frequencies represent the number of assessors who associated each CATA attribute with the analyzed samples, while the *p*-values indicate the existence of sensory differences between samples. The sensory attributes that significantly contributed to sample discrimination (*p* < 0.05) were uneven color, high moisture content, rich and smooth mouthfeel, dry mouthfeel, low fish odor intensity, fresh orange aroma, balanced fish–orange aroma, low fish flavor intensity, high fish flavor intensity, dominant fish flavor, balanced fish–citrus flavor, and freshness perception. The variable selection frequencies of these attributes indicate distinct panelist perceptions regarding the visual, olfactory, gustatory, and textural characteristics of the samples. In contrast, the attributes light color, visual homogeneity, visible granularity, perceptible granularity, creamy texture, high fish odor intensity, oxidized/rancid odor, bitter taste, perceptible astringency, long-lasting aftertaste, and unpleasant aftertaste showed a more uniform perception among samples, with no statistically significant differences observed (*p* > 0.05).

**Figure 3:**
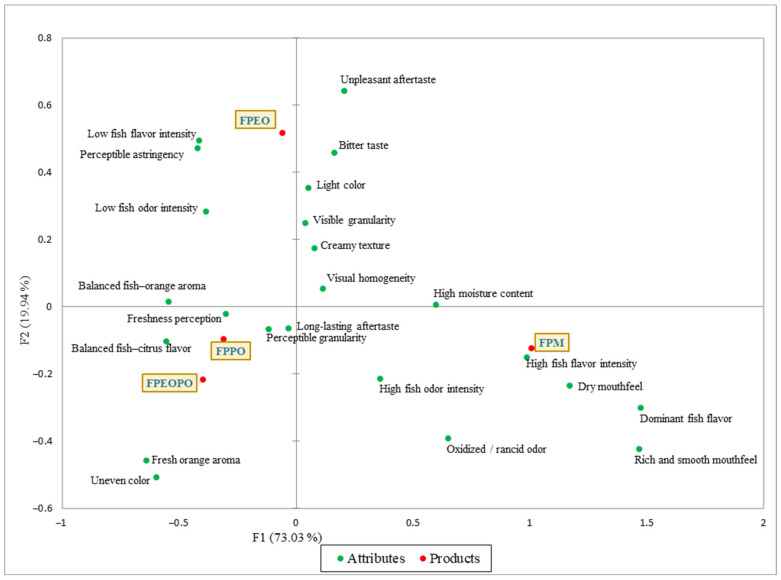
Biplot diagram of correspondence analysis (CA) of the CATA attributes of the samples studied (F1 + F2 = 92.97%). FPM—control spreadable fish paste without orange-derived ingredients; FPEO—spreadable fish paste formulated with orange extract; FPPO—spreadable fish paste formulated with orange peel; FPEOPO—spreadable fish paste formulated with a combination of orange extract and orange peel. Values represent mean sensory scores obtained using a 9-point hedonic scale, where higher values indicate greater sensory acceptance.

The CATA biplot obtained by correspondence analysis (CA) illustrates the relationships between the fish paste samples and their specific sensory attributes (Figure 3). The factorial axes of the biplot (F1 and F2) jointly explain 92.97% of the total variance, indicating a good representation of the data within this two-dimensional space. The positioning of samples in close proximity to specific attributes reflects the dominant sensory associations for each product, in agreement with the selection frequencies and the results of Cochran’s Q test. The FPM sample (control) is positioned close to attributes such as high fish flavor intensity, dominant fish flavor, dry mouthfeel, rich and smooth mouthfeel, and high moisture content, indicating that these characteristics define its sensory profile. In contrast, the FPEO sample (fish paste with orange extract) is characterized by attributes including low fish flavor intensity, perceptible astringency, low fish odor intensity, unpleasant aftertaste, bitter taste, and light color. This distribution suggests that the addition of orange extract leads to an imbalance in the aromatic and gustatory profile, largely masking the characteristic fish flavor and aroma. The FPPO sample (fish paste with orange peel) and the FPEOPO sample (fish paste with a combination of orange peel and orange extract) are located in very close proximity to each other, indicating that they were perceived by consumers as having similar sensory characteristics. These samples are mainly associated with attributes such as balanced fish–citrus flavor, freshness perception, perceptible granularity, long-lasting aftertaste, balanced fish–orange aroma, and fresh orange aroma, highlighting a more harmonious integration of citrus-related sensory notes.

Attributes such as visual homogeneity and creamy texture, positioned between the FPM, FPPO, and FPEOPO samples, indicate that these characteristics are common to all three products, reflecting their sensory similarities. As observed, the FPPO and FPEOPO samples exhibit the greatest similarity to the control sample (FPM) in terms of sensory attributes, a finding that is consistent with the hedonic results (Figure 2), where a comparable level of consumer preference was observed for these samples, particularly with respect to overall liking.

#### 3.5.3. Quantitative Descriptive Analysis (QDA)

Within the QDA analysis, 18 sensory attributes were evaluated using an intensity scale ranging from 1 to 9 (Table 11). These attributes were established by the authors of the study in order to provide a controlled and reproducible description of the sensory profile of the analyzed products. For this reason, the set of attributes used in the QDA differs from that applied in the CATA test, where the descriptors were selected by the sensory panel and reflected the spontaneous perceptions of the evaluators.

The results of the QDA revealed statistically significant differences (*p* < 0.05) for only 3 out of the 18 sensory attributes across the four analyzed fish paste samples, namely: fish odor intensity, presence of citrus notes, and perceived citrus flavor. For the remaining sensory attributes, no statistically significant differences were observed (*p* > 0.05). In contrast, the global QDA analysis considering the effects of sample, sensory attribute, and their interaction showed significant differences among sensory attributes (*p* < 0.0001), among samples (*p* < 0.008), as well as for their interaction (*p* < 0.0001). This outcome can be explained by the fact that the global analysis accounts for the overall variability across attributes, samples, and their interaction, whereas the analysis of each individual sensory attribute examines only the differences among fish paste samples for that specific attribute. Consequently, variability at the individual attribute level is reduced, and significant differences are limited, as observed, to those sensory attributes directly influenced by product formulation, namely the addition of citrus-derived ingredients and their interaction with the fish matrix, while the remaining characteristics remained relatively similar across formulations.

#### 3.5.4. Principal Component Analysis (PCA)

The PCA, performed based on the results of the QDA analysis and presented in Figure 3, illustrates the relationships between the analyzed sensory attributes and the studied fish paste samples, as well as the differentiation of their sensory profiles. The first two principal components (F1 and F2) explain a high proportion of the total sensory data variability (90.28%), indicating an adequate representation of the sensory profiles of the fish paste samples within this reduced dimensional space.

The FPM sample (control) is associated with sensory attributes such as paste shininess, presence of off-odors, paste homogeneity, fish flavor intensity, and fish odor intensity. In addition, both the FPM and FPEO samples are characterized by smoothness and adhesiveness. Beyond these attributes, the FPEO sample is further characterized by the presence of citrus notes, which were most perceptible in this formulation, as also reflected by the highest mean value reported in Table 2 for this attribute (5.67 ± 1.83) as well as by sourness, presence of air bubbles, sweetness, and perceived citrus taste. These findings indicate that the FPEO sample exhibits a sensory profile strongly influenced by citrus-related attributes, suggesting that orange extract exerts a greater impact on modifying the sensory profile than orange peel powder. In comparison, the FPPO sample (fish paste with orange peel powder) is characterized by attributes such as a more intense color, color uniformity, a more pronounced aftertaste, slight bitterness, and a slightly higher viscosity than the other samples. This observation is consistent with the results reported in Table 2, where viscosity reached its highest mean value for this sample (5.33 ± 0.92). The FPEOPO sample is positioned in an intermediate location between the two citrus-added samples (FPEO and FPPO), but closer to FPPO, indicating a sensory profile more similar to that of the orange peel formulation.

**Figure 4:**
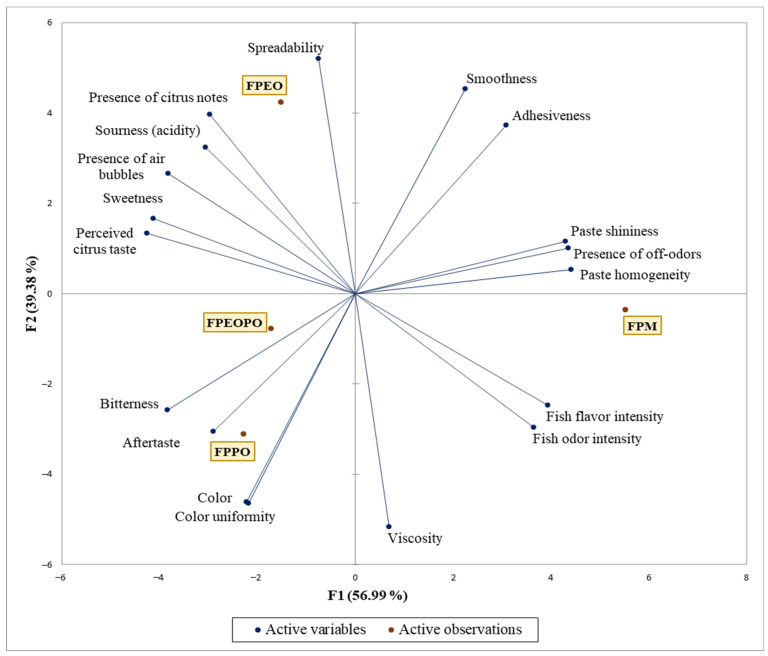
Distribution of fish paste samples according to QDA sensory attributes using principal component analysis. FPM—control spreadable fish paste without orange-derived ingredients; FPEO—spreadable fish paste formulated with orange extract; FPPO—spreadable fish paste formulated with orange peel; FPEOPO—spreadable fish paste formulated with a combination of orange extract and orange peel.

Moreover, the positioning of the FPEOPO sample highlights a well-balanced sensory profile, defined by a moderate intensity of the citrus notes, without dominating the other organoleptic attributes. This harmonization of sensory components suggests an effective integration of the bioingredient into the product matrix, helping to maintain a pleasant and coherent taste character. At the same time, this balance is also reflected in the results of the hedonic evaluation, where FPEOPO recorded the highest overall acceptability score (“overall liking”), indicating a clear preference from the evaluators. Therefore, the correlation between sensory profile and consumer response supports the potential of this variant to meet current functional food requirements with optimized sensory properties (Figure 4).

#### 3.5.5. External Preference Mapping (PrefMap)

In the context of applied food product research, identifying how consumers are segmented into distinct preference groups has become increasingly important, rather than relying solely on the determination of an overall mean preference. This approach is justified by the growing competitiveness of the market and the diversification of consumer demands, as product success depends on its ability to meet the preferences of specific consumer segments, rather than being uniformly preferred by all consumers [64,65]. Within this framework, external preference mapping (PrefMap) was applied to analyze the structure of consumer preferences and their relationship with the evaluated fish paste samples.

**Figure 5:**
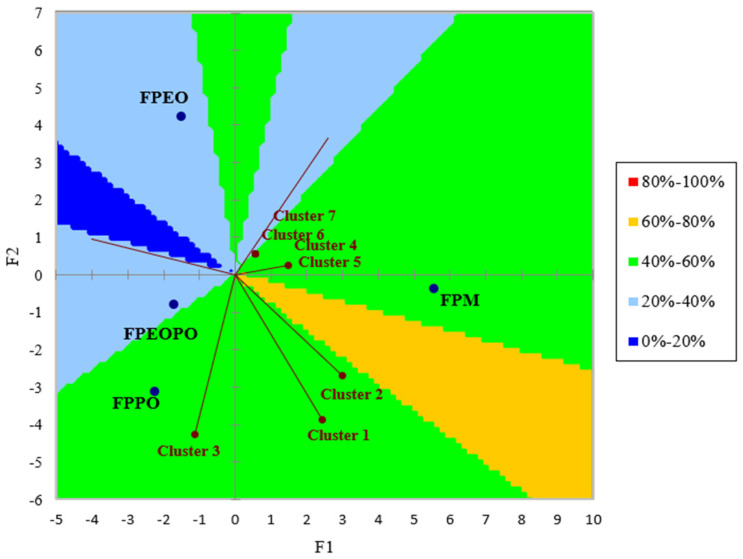
External preference map (PrefMap) of fish paste samples based on sensory PCA space. FPM—spreadable fish paste without orange-derived ingredients; FPEO—spreadable fish paste formulated with orange extract; FPPO—spreadable fish paste formulated with orange peel; FPEOPO—spreadable fish paste formulated with a combination of orange extract and orange peel.

Figure 5 presents the graphical representation of the external preference mapping, illustrating the distribution of fish paste samples relative to the seven consumer clusters identified within the tasting panel of 24 evaluators, as well as the preference regions, indicated by different colors. The results reveal a clear heterogeneity of preferences, with panelists divided into seven distinct clusters, each associated with different preference directions. The FPM and FPPO samples are positioned in regions corresponding to higher preference levels for most of the identified clusters, whereas the FPEO and FPEOPO samples are generally associated with regions reflecting lower preference levels. Nevertheless, the FPEOPO sample is located in close proximity to the preference region associated with FPM and FPPO, being positioned closest to FPPO, which suggests a strong sensory relationship between these samples. Although FPEOPO achieved the highest scores in the hedonic evaluation (overall liking), this outcome can be explained by its previously identified balanced sensory profile, which favors high global acceptability. In contrast, within the external preference mapping—where preferences are analyzed across distinct evaluator clusters—FPEOPO is not associated with the areas of maximum preference. This is because these regions are correlated with a more pronounced intensity of certain dominant sensory attributes. Consequently, the results indicate that while FPEOPO is the most accepted sample at the hedonic level, it is not the most preferred product in a directional sense, where preferences are driven by specific sensory attributes and consumer segmentation.

This method also presents certain limitations, as the analysis is typically based on the first two dimensions of the sensory space used for graphical representation, without certainty that these dimensions fully capture the factors driving consumer liking [33]. In this context, the fact that FPEOPO, despite achieving the highest hedonic scores, is not located within the maximum preference regions of the PrefMap suggests that overall consumer preference is not exclusively explained by the dimensions included in the map but may also be influenced by additional sensory dimensions that are not represented in the two-dimensional space.

The sensory evaluation results obtained through hedonic analysis, CATA, QDA, PCA, and PrefMap are complementary and indicate that the addition of citrus-derived ingredients (orange) influences the sensory perception of fish pastes in a differentiated manner (PrefMap—Figure 5), but positively in most cases (hedonic analysis—Figure 2), depending on the type of addition (orange extract or orange peel powder). Hedonic analysis (Figure 2) showed that samples containing orange peel powder (FPPO) and the combination of orange extract and orange peel powder (FPEOPO), the latter achieving the highest overall liking score, were perceived as sensorially pleasant and superior to the control sample (FPM), demonstrating good consumer acceptability of citrus-based additions.

Descriptive analyses (CATA—Figure 3 and QDA—Table 11) revealed that the addition of orange extract induced more pronounced changes in the olfactory and gustatory profile, whereas orange peel powder led to notable but less divergent sensory modifications compared to the control, relative to those observed for the extract. The PCA (Figure 4) highlighted the relationships between fish paste samples and dominant sensory attributes, enabling visualization of sensory profile differentiation within the space defined by the first two principal components. The intermediate positioning of FPEOPO, closer to FPPO than to FPEO, indicates a balanced sensory profile, characterized by a moderate influence of citrus notes while preserving the typical characteristics of fish paste. This distribution suggests that combining orange extract with orange peel powder mitigates the intense citrus impact observed in FPEO, resulting in a more harmonious sensory profile, consistent with the high hedonic scores obtained for this sample.

The external preference mapping (Figure 5) demonstrated a clear segmentation of consumers, with preferences distributed across distinct clusters and associated with specific dominant sensory attributes. Although FPEOPO exhibited high overall acceptability, it was not consistently positioned within the zones of maximum preference, being associated with variable preference levels depending on evaluator distribution. These findings indicate that samples containing citrus ingredients differ mainly in how specific sensory attributes are perceived, and that the combination of orange extract and orange peel powder (FPEOPO) leads to a more balanced sensory profile, reflected by high hedonic scores as well as an intermediate positioning in both PCA and external preference mapping. The observed differences between global acceptability and cluster-based preferences highlight that the sensory perception of fish pastes is differentially influenced by the type of citrus addition used.

## 4. Conclusions

Fish-based foods are recognized for their significant role in a balanced diet and promoting good health. This study aimed to create and evaluate a product made from *Cyprinus carpio* fillets, enhanced with natural ingredients from orange peel. The research used simple methods and natural ingredients, following current trends. The results indicate that the incorporation of orange peel extract (OE) and orange peel powder (PPO) enhanced the bioactive profile and antioxidant activity of the product, while maintaining its overall nutritional composition. Specifically, OE + PPO was associated with increased antioxidant activity, a factor of particular relevance given the susceptibility of fish products to oxidative processes. These biocomponents also influenced the physicochemical and color parameters, helping to define a distinct sensory profile, correlated with the composition of the product. From a microbiological point of view, the absence of *Escherichia coli* and *Staphylococcus aureus* confirms the safety of the product under experimental conditions, while the dynamics of mold development suggest that complex interactions between bioactive compounds and the food matrix may influence stability during storage. *E. coli* and *S. aureus* were absent throughout the tests, and other factors than the presence of flavonoids and polyphenols may have influenced the development of molds in the fish paste where the mixture of orange peel extract and orange peel powder was used. The acceptability of fish-based products is often limited by the perception of smell, which is one of the main determinants of consumer rejection. In this context, the sensory evaluation highlighted that the citrus-enriched samples differed mainly through the perception mode of the specific sensory attributes. This formulation proposes a viable alternative to mitigate this limitation, helping to improve the sensory acceptability of the product. The combination of OE and PPO resulted in a more balanced sensory profile, supported by higher hedonic scores and an intermediate positioning in both main component analysis (PCA) and external preference mapping. Overall, the study highlights the applicative potential of the use of citrus byproducts as sources of bioactive compounds in the formulation of fish-based products, contributing to the development of products with superior nutritional value and increased relevance for both industry and consumers. At the same time, the compositional variability of the commercial extracts used remains an important aspect to be taken into account, and further investigations including a detailed characterization of bioactive compounds are needed to consolidate the results.

## Figures and Tables

**Table 1:** Heat treatment programs for fish fillets.

Stage	Time (min)	Temperature (°C)	Temperature in the Centre of the Product (°C)	Humidity Within the Equipment (%)
Drying	30	40	35	40
Smoking	30	65	50	40
Boiling	20	76	72	99
Drying	05	72	65	40

**Table 2:** Experimental samples studied and their components.

Samples	Biocomponent	Biocomponent %	Raw Material Quantity (g)
FPM	-	-	400
FPEO	Liquid extract from orange peel	1	400
FPPO	Orange peel powder	1	400
FPEOPO	Liquid extract from orange peel + Orange peel powder	0.5 + 0.5	400

FPM: control sample spreadable paste made from fish fillets (*Cyprinus carpio*); FPEO: experimental sample 1—spreadable paste made from fish fillets (*Cyprinus carpio*) supplemented with liquid extract from orange peels; FPPO: experimental sample 2—spreadable paste made from fish fillets (*Cyprinus carpio*) supplemented with orange peel powder; FPEOPO: experimental sample 3—spreadable paste made from fish fillets (*Cyprinus carpio*) supplemented with liquid extract from orange peel + orange peel powder.

**Table 3:** Sensory categories and corresponding CATA terms used in the study.

Sensory Attributes	CATA Terms
Appearance	light color, uneven color, visual homogeneity, visible granularity
Olfactive perception	low fish odor intensity, high fish odor intensity, fresh orange aroma, balanced fish–orange aroma, oxidized/rancid odor
Taste	low fish flavor intensity, high fish flavor intensity, dominant fish flavor, balanced fish–citrus flavor, bitter taste, perceptible astringency, perceptible astringency, long-lasting aftertaste, unpleasant aftertaste, freshness perception
Texture	perceptible granularity, creamy texture, high moisture content, rich and smooth mouthfeel, dry mouthfeel

**Table 4:** Sensory attributes and their classification into main sensory modalities used for QDA.

Main Sensory Modalities	Sensory Attributes
Appearance	color, color uniformity, paste homogeneity, presence of air bubbles, paste shininess
Aroma	fish odor intensity, presence of citrus notes, presence of off-odors
Taste	fish flavor intensity, perceived citrus taste, sweetness, bitterness, sourness (acidity), aftertaste
Texture	viscosity, smoothness, adhesiveness, spreadability

**Table 5:** Chemical composition of the samples analyzed.

	FPM	FPEO	FPPO	FPEOPO
Total Protein, %	16.25 ± 0.82 ^b^	16.96 ± 1.55 ^b^	15.03 ± 0.83 ^a^	17.05 ± 0.48 ^c^
Lipid, %	8.1 ± 0.25 ^d^	7.21 ± 0.09 ^b^	7.75 ± 0.15 ^c^	6.87 ± 0.11 ^a^
Ash, %	1.84 ± 0.11 ^a^	1.82 ± 0.03 ^a^	1.9 ± 0.06 ^a^	1.93 ± 0.03 ^a^
Dry Matter, %	27.21 ± 0.07 ^b^	28.78 ± 0.05 ^c^	26.62 ± 0.21 ^a^	28.63 ± 0.09 ^c^
Humidity, %	72.8 ± 0.04 ^c^	70.23 ± 0.02 ^a^	73.59 ± 0.04 ^d^	71.4 ± 0.06 ^b^

Superscript letters (^a^, ^b^, ^c^, ^d^) indicate statistically significant differences between the groups in each row based on the unidirectional ANOVA test followed by Tukey’s HSD post hoc test (*p* < 0. 05). Groups that do not have the same letter in the same row are significantly different.

**Table 6:** Characterization of bioactive compounds, antioxidant activity and colorimetric parameters of orange peel powder (OP) and orange peel extract (EO).

Parameters	Orange Powder (PPO)	Extract (EO)
Total Carotenoids, mg/100 g dw	2.06 ± 0.00	1.44 ± 0.01
β-caroten (mg/100 g dw)	1.53 ± 0.001	0.76 ± 0.01
Licopen (mg/100 g dw)	0.31 ± 0.001	0.10 ± 0.00
Total Flavonoids, mg CE/100 g dw	3.88 ± 0.121	1.23 ± 0.018
Total Polyphenols, mg GAE/100 g dw	12.78 ± 0.01	8.53 ± 0.12
DPPH, µmol TE/g dw	14.13 ± 0.03	13.65 ± 0.48
Inhibition %	93.60 ± 0.20	90.20 ± 0.41
L*	79.19 ± 0.62	77.80 ± 0.20
a*	4.28 ± 0.15	1.37 ± 0.36
b*	33.32 ± 0.21	17.68 ± 1.40
C* (Chroma)	33.60 ± 0.26	17.73 ± 1.43
H* (Hue angle)	82.68 ± 0.26	86.05 ± 0.83
∆E	86.03 ± 0.59	80.21 ± 0.11

**Table 7:** Phytochemical characterization and antioxidant activity of orange peel powder (PPO) and orange peel extract (EO) in experimental samples from fish fillets (*Cyprinus carpio*).

Phytochemical Characteristics and Antioxidant Activity	FPM	FPEO	FPPO	PFEOPO
Total Carotenoids, mg/100 g dw	-	0.32 ± 0.00 ^a^	0.45 ± 0.01 ^c^	0.35 ± 0.005 ^b^
β-caroten (mg/100 g dw)	-	0.23 ± 0.01 ^a^	0.31 ± 0.004 ^c^	0.26 ± 0.005 ^b^
Licopen (mg/100 g dw)	-	0.04 ± 0.005 ^a^	0.09 ± 0.003 ^c^	0.06 ± 0.003 ^b^
Total Flavonoids, mg CE/100 g dw	0.38 ± 0.00 ^a^	0.604 ± 0.00 ^b^	1.025 ± 0.030 ^d^	0.825 ± 0.01 ^c^
Total Polyphenols, mg GAE/100 g dw	0.63 ± 0.01 ^a^	0.93 ± 0.01 ^b^	1.31 ± 0.02 ^d^	1.17 ± 0.02 ^c^
DPPH, µmol TE/g dw	4.5 ± 0.04 ^a^	7.60 ± 0.14 ^b^	12.14 ± 0.17 ^d^	10.28 ± 0.12 ^c^
Inhibition %	8.63 ± 0.21 ^a^	30.46 ± 0.29 ^d^	19.26 ± 0.28 ^b^	25.19 ± 0.14 ^c^

Superscript letters (^a^, ^b^, ^c^, ^d^) indicate statistically significant differences between the groups in each row based on the unidirectional ANOVA test followed by Tukey’s HSD post hoc test (*p* < 0. 05). Groups that do not have the same letter in the same row are significantly different.

**Table 8:** Analysis of chromatic indicators of the analyzed samples.

	FPM	FPEO	FPPO	PFEOPO
L* (D65)	67.144 ± 0.513 ^b^	68.44 ± 0.419 ^c^	66.432 ± 0.513 ^a^	68.018 ± 0.150 ^c^
a* (D65)	6.124 ± 0.01 ^c^	5.844 ± 0.03 ^b^	5.47 ± 0.07 ^a^	5.55 ± 0.07 ^a^
b* (D65)	14.092 ± 0.05 ^a^	14.302 ± 0.02 ^a^	15.082 ± 0.01 ^b^	15.028 ± 0.11 ^b^
C* (Chroma)	15.349 ± 0.06 ^a^	15.428 ± 0.01 ^a^	16.043 ± 0.122 ^b^	16.020 ± 0.134 ^b^
H* (Hue Angle)	66.648 ± 0.139 ^a^	67.802 ± 0.183 ^b^	70.065 ± 0.148 ^d^	69.731 ± 0.122 ^c^
∆E	68.915 ± 0.450 ^a^	70.166 ± 0.418 ^d^	68.341 ± 0.501 ^a^	69.879 ± 0.141 ^b^

Superscript letters (^a^, ^b^, ^c^, ^d^) indicate statistically significant differences between the groups in each row based on the unidirectional ANOVA test followed by Tukey’s HSD post hoc test (*p* < 0. 05). Groups that do not have the same letter in the same row are significantly different.

**Table 9:** Bacterial and mold levels in tested samples at different time points over 7 days of storage.

Microorganism	Storage Day	Sample			
		FPM	FPEO	FPPO	FPEOPO
*E. coli*	0	nd	nd	nd	nd
	3	nd	nd	nd	nd
	7	nd	nd	nd	nd
*S. aureus*	0	nd	nd	nd	nd
	3	nd	nd	nd	nd
	7	nd	nd	nd	nd
Molds, log CFU·g^−1^	0	2.15 ± 0.21 ^c^	1 ± 0.70 ^b^	2.54 ± 0.08 ^d^	0 ± 0 ^a^
	3	2.48 ± 0 ^b^	0 ± 0 ^a^	2.40 ± 0.42 ^b^	0 ± 0 ^a^
	7	3.01 ± 0.14 ^d^	0 ± 0 ^a^	2.97 ± 0.10 ^c^	2.15 ± 0.21 ^b^

Superscript letters (^a^, ^b^, ^c^, ^d^) indicate statistically significant differences between the groups in each row based on the unidirectional ANOVA test followed by Tukey’s HSD post hoc test (*p* < 0. 05). “nd” = not detected.

**Table 10:** Selection frequency of CATA attributes and Cochran’s Q test *p*-values for fish paste samples.

Attributes	Selection Frequency of CATA Attributes	Cochran’s Q Test *p*-Values
Light color	30	0.253
Uneven color	50	<0.0001
Visual homogeneity	95	0.392
Visible granularity	10	0.511
Perceptible granularity	12	0.066
Creamy texture	75	0.147
High moisture content	29	0.003
Rich and smooth mouthfeel	6	0.005
Dry mouthfeel	10	0.004
Low fish odor intensity	32	0.002
High fish odor intensity	16	0.225
Fresh orange aroma	37	<0.0001
Balanced fish–orange aroma	21	<0.0001
Oxidized/rancid odor	2	0.572
Low fish flavor intensity	23	0.006
High fish flavor intensity	21	0.000
Dominant fish flavor	29	<0.0001
Balanced fish–citrus flavor	37	<0.0001
Bitter taste	17	0.297
Perceptible astringency	7	0.392
Long-lasting aftertaste	34	0.129
Unpleasant aftertaste	13	0.078
Freshness perception	64	<0.0001

**Table 11:** Mean sensory scores (±SD) of fish paste samples and significance of sample, sensory attribute, and their interaction.

Sensory Attributes	FPM	FPEO	FPPO	FPEOPO	*p*-Value
Color	4.83 ± 1.37	4.71 ± 1.63	5.46 ± 1.67	5.25 ± 1.42	0.290
Color uniformity	8.42 ± 0.88	8.38 ± 0.88	8.63 ± 0.65	8.54 ± 0.66	0.664
Paste homogeneity	8.58 ± 0.65	8.25 ± 0.74	8.17 ± 0.76	8.21 ± 0.72	0.176
Presence of air bubbles	3.00 ± 1.91	3.83 ± 1.52	3.46 ± 1.28	3.63 ± 1.24	0.274
Paste shininess	5.25 ± 1.67	4.83 ± 1.27	4.63 ± 1.17	4.79 ± 1.35	0.450
Fish odor intensity	5.17 ± 1.81	3.25 ± 1.70	4.25 ± 1.92	3.75 ± 1.67	0.003
Presence of citrus notes	1.67 ± 0.96	5.67 ± 1.83	2.96 ± 1.65	3.63 ± 1.55	0.000
Presence of off-odors	2.04 ± 1.40	1.75 ± 1.15	1.63 ± 1.10	1.71 ± 1.08	0.644
Fish flavor intensity	5.08 ± 2.08	4.00 ± 2.15	4.42 ± 1.53	4.33 ± 1.43	0.225
Perceived citrus taste	1.33 ± 0.56	4.08 ± 2.60	3.67 ± 1.40	3.38 ± 1.21	0.000
Sweetness	2.42 ± 1.28	3.25 ± 1.92	3.00 ± 1.59	3.21 ± 1.28	0.222
Bitterness	1.46 ± 0.78	1.75 ± 0.94	2.17 ± 1.01	1.92 ± 0.83	0.053
Sourness (acidity)	1.67 ± 0.87	2.38 ± 1.74	2.04 ± 1.37	1.88 ± 1.19	0.308
Aftertaste	4.13 ± 2.63	4.17 ± 1.83	4.42 ± 2.12	4.54 ± 1.64	0.883
Viscosity	5.21 ± 1.80	4.92 ± 1.21	5.33 ± 0.92	5.13 ± 1.03	0.721
Smoothness	7.75 ± 1.15	7.88 ± 0.99	7.17 ± 1.40	7.29 ± 1.08	0.107
Adhesiveness	4.08 ± 2.21	4.00 ± 1.14	3.54 ± 0.93	3.79 ± 1.02	0.558
Spreadability	6.83 ± 1.17	7.17 ± 1.13	6.71 ± 1.46	6.92 ± 1.21	0.632
Effects of sample, sensory attribute, and their interaction					*p*-value
Sensory attributes					<0.0001
Fish paste samples					0.008
Sensory attributes × Fish paste samples					<0.0001

FPM—control spreadable fish paste without orange-derived ingredients; FPEO—spreadable fish paste formulated with orange extract; FPPO—spreadable fish paste formulated with orange peel; FPEOPO—spreadable fish paste formulated with a combination of orange extract and orange peel. Values represent mean sensory scores obtained using a 9-point hedonic scale, where higher values indicate greater sensory acceptance.

## Data Availability

The original contributions presented in the study are included in the article; further inquiries can be directed to the corresponding author.
